# The effect of reusing wipes for particle collection

**DOI:** 10.1007/s12127-015-0185-9

**Published:** 2015-11-04

**Authors:** Jessica L. Staymates, Matthew E. Staymates, Jeffrey Lawrence

**Affiliations:** National Institute of Standards and Technology (NIST), 100 Bureau Drive, Mailstop 8371, Gaithersburg, MD 20899 USA

**Keywords:** Trace detection, Particle collection, Swiping, Explosives, Narcotics, Ion mobility spectrometry

## Abstract

Sample collection for Ion Mobility Spectrometry (IMS) analysis is typically completed by swiping a collection wipe over a suspect surface to collect trace residues. The work presented here addresses the need for a method to measure the collection efficiency performance of surface wipe materials as a function of the number of times a wipe is used to interrogate a surface. The primary purpose of this study is to investigate the effect of wipe reuse, i.e., the number of times a wipe is swiped across a surface, on the overall particle collection and IMS response. Two types of collection wipes (Teflon coated fiberglass and Nomex) were examined by swiping multiple times, ranging from 0 to 1000, over representative surfaces that are common to security screening environments. Particle collection efficiencies were determined by fluorescence microscopy and particle counting techniques, and were shown to improve dramatically with increased number of swiping cycles. Ion mobility spectrometry was used to evaluate the chemical response of known masses of explosives (deposited after reusing wipes) as a function of the wipe reuse number. Results show that chemical response can be negatively affected, and greatly depends upon the conditions of the surface in which the wipe is interrogating. For most parameters tested, the PCE increased after the wipe was reused several times. Swiping a dusty cardboard surface multiple times also caused an increase in particle collection efficiency but a decrease in IMS response. Scanning electron microscopy images revealed significant surface degradation of the wipes on dusty cardboard at the micrometer spatial scale level for Teflon coated wipes. Additionally, several samples were evaluated by including a seven second thermal desorption cycle at 235°C into each swipe sampling interval in order to represent the IMS heating cycle. Results were similar to studies conducted without this heating cycle, suggesting that the primary mechanism for wipe deterioration is mechanical rather than thermal.

## Introduction

Swipe sampling is a common method for collecting particles for environmental sampling and forensic applications. This physical swiping of surfaces is also used at airports and other security settings to screen people and their belongings for explosive materials that could suggest the presence of potential terrorist threats [4, 24]. The screening technique that commonly utilizes dry wipe sampling in these security settings is ion mobility spectrometry (IMS), which is often chosen due to its sensitivity, rapid analysis time and ease of use. While biological and environmental screening often uses wet wipes for collection and then extraction for an analytical procedure [3, 14, 15], the wipes for IMS screening must be dry to avoid chemical interferences with the IMS technique [5]. In the last decade, there have been tens of thousands of these instruments deployed worldwide in airports alone [4]. IMS is also used to screen for illicit narcotics, therefore this technology is also used for screening visitors and mail at prisons [2], detecting potential drug smuggling activities [6, 17], and for medical and biological purposes [1, 18].

The wipe material used to collect trace contamination varies among different instruments and their manufacturers [22]. Since the chemical analysis includes heating the wipe with collected material to temperatures exceeding 200 °C, it is important that the wipes are thermally stable and have low chemical background. The wipes can be a costly consumable, and with many thousands of people and their belongings being screened each day, it is common practice to reuse the wipe material as long as no threat has previously been detected with it. Different guidelines exist for when the wipe is no longer usable, such as after 10 to 20 uses or as long as it is not visibly dirty or damaged [8, 13]. But little research has been done to examine the number of times and under what conditions these different wipe materials can actually be reused and remain effective in terms of retaining particle collection capabilities and IMS response.

The goal of this study was to investigate the aforementioned subject of reusing wipes. Two common wipe materials were studied, Nomex and Teflon^1^ -coated fiberglass (TCFG), using a robotic arm to swipe the wipe over a surface multiple times. Ideal conditions were tested using clean surfaces. To mimic field conditions similar to surfaces that would be potentially screened at a security checkpoint, some surfaces were doped with standard dust [10, 11]. For some studies, the wipe was inserted into a thermal desorber during each swiping cycle to mimic the heating and cooling cycle that a wipe would encounter during IMS analysis. After multiple swipes and thermal desorption cycles, the wipes were evaluated for particle collection efficiency and the IMS response. The data and results of the effects of reusing wipe sampling media are discussed here.

## Materials and methods

### Wipe testing system

**Fig. 1:**
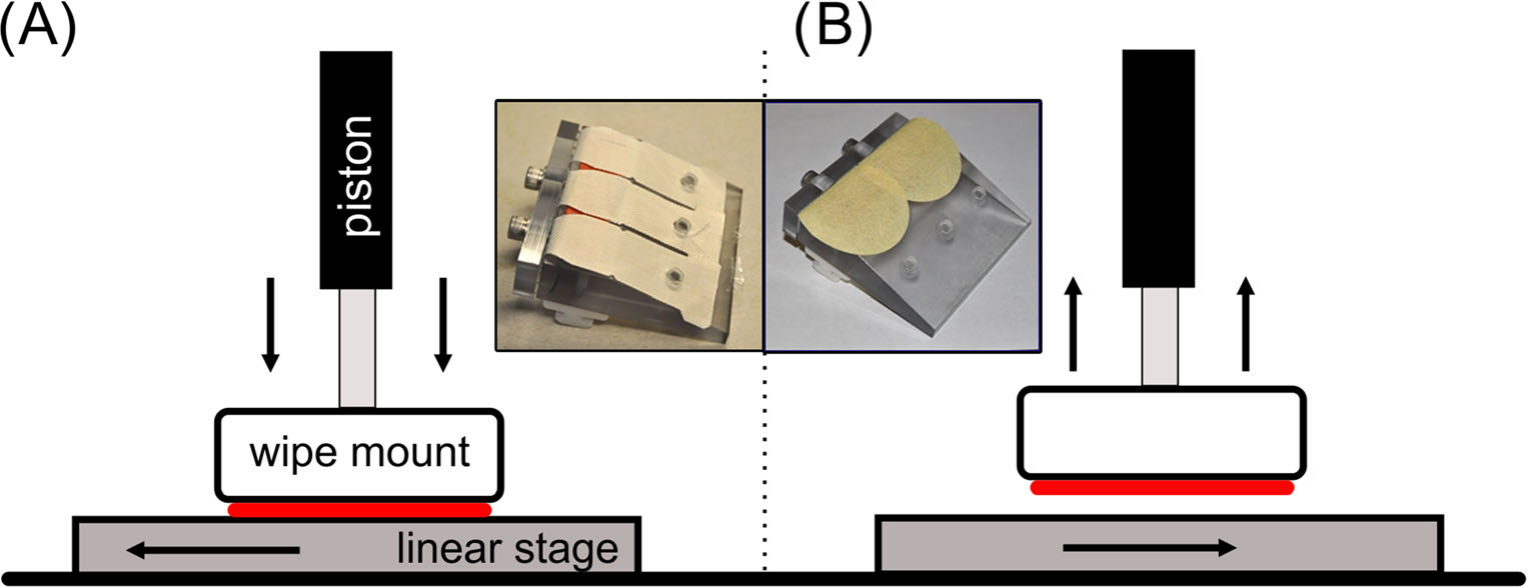
Schematic diagram illustrating the operation of the wipe swiping system. The wipe material is represented by the *red line*. In **a**, the piston drives the wipe mount onto the linear stage which then translates horizontally at a constant velocity. Once the desired distance has been reached, the piston releases the wipe mount and the stage returns to its original position, shown in (**b**). The inset images show two examples of wipe mount designs. The left image holds three TCFG wipes and the right image holds two Nomex wipes

Wipes were reused (swiped repeatedly over a surface) with a system consisting of a custom built linear actuator and pneumatic piston, and a custom LabVIEW control code (National Instruments, Austin TX). A unique mounting head, fabricated on a 3D printer (Objet 30 Pro, Stratasys Inc.), enabled up to three individual wipes to be positioned above the translating substrate. The mounting head was fastened to a pneumatic piston that provided the necessary force between the surface and the wipes. Air pressure was used to drive the wipes (secured in the mounting head) onto a surface, then the linear actuator was translated underneath to mimic a swiping motion with a repeatable force, distance, and velocity. A schematic diagram of the setup, along with inset photographs of two different mounting heads, is given in Fig. 1. The system was fully automated, other than requiring the user to mount the sample wipes and input the number of times to swipe over a surface. Confirmation of the applied force provided by the pneumatic piston was performed with a Tekscan model 5101 array-based force sensing resistor (Tekscan Inc. South Boston, MA). The air pressure was adjusted such that the downward force exerted by the wipe material was approximately seven Newtons (N), a magnitude previously found to be the average force a user would apply to a surface when asked to use “firm” force [23]. A swiping distance of 10 cm was chosen to be consistent with previous experiments [23], and velocity of 70 mm/s was designated based on velocity measurements of a series of volunteers wiping naturally along a surface (unpublished NIST data).

### Wiping materials and surfaces

TCFG wipes and Nomex wipes were chosen for this study because of their relevance to checkpoint security – the majority of commercially available IMS trace detectors currently deployed at major US airports utilize Nomex and TCFG swabs [12]. Other materials less commonly used for IMS include metal mesh filters, muslin cloth, and certain types of paper. The particle collection characteristics and low chemical background make these materials a popular choice for IMS-based trace contraband detection. Sampling surfaces used in this study included a woven canvas material typically sampled from luggage, along with cardboard samples cut from unused cardboard boxes. Dusty cardboard samples were prepared by adding organic contaminants from household dust (NIST SRM # 2585, organic contaminants in house dust, [10]) to simulate representative surfaces encountered in cargo facilities and passenger checkpoints. Dust was mixed with water to form a suspension in a dropper bottle, then deposited onto the cardboard blanks and allowed to dry. During the swiping process, each wipe came into contact with 4 to 6 spots of dust containing nominally 160 μg of dust per drop. Cardboard surfaces with fresh dust deposits were replenished several times throughout the process of reusing wipes to represent new surfaces that would be interrogated during a checkpoint screening process. All cardboard surfaces were used for a maximum of 100 swiping passes.

Wipes were reused 10, 50, 100, 500, and 1000 times by swiping over each surface in a single direction with a distance of 10 cm and an average force of 7 N. Wipes were then carefully removed from the swiping mount. Particle collection efficiencies (PCE) were later measured by counting 39 μm diameter fluorescent polymer microspheres dry deposited on vinyl surfaces before swipe sampling and comparing that to the number of the particles collected on the wipe after swipe sampling, as discussed in detail in other work [16, 22]. Briefly, PCE was calculated by dividing the number of particles collected on the wipe by the number of particles that were originally on the vinyl surface, and multiplying that number by 100 to calculate a percent. Next, precise amounts of cyclotrimethylenetrinitramine (RDX) explosive were deposited directly onto each used wipe by a piezoelectric inkjet printing process [20, 21, 24]. The mass of deposited RDX is not reported here for security purposes. The mass was within the linear dynamic range of the instrument and of sufficient level to elicit a positive alarm response 95 % of the time. The RDX-doped wipes were then analyzed with IMS to determine whether the repetitive use of the wipe affected the IMS response. TCFG wipes were analyzed with an Itemiser DX IMS instrument (Morpho Detection, Andover, MA), and the Nomex wipes were analyzed with an Ionscan 500DT IMS instrument (Smith’s Detection, Edgewood, MD). The PCE and IMS response were also determined for ten control wipes that were not swiped. All swiping conditions were performed in replicates of ten. It is important to note that the explosive was not collected by swiping; all samples were repetitively swiped first, then evaluated for PCE using fluorescent particles, and then evaluated separately for IMS response of RDX from direct inkjet deposition. The purpose of the IMS analysis was to determine whether there was a change in response due to the physical condition of the wipe.

### Scanning electron microscopy

The scanning electron microscope (SEM) used in this work was an FEI Company (Hillsboro, OR) Quanta 200F environmental scanning electron microscope (SEM). 10 mm by 10 mm sections were cut from the reused wipes and mounted on imaging stubs with carbon tape. Samples were coated with 10 nm of gold to prevent sample charging and facilitate enhanced imaging of the wipe surface. All samples were analyzed in high vacuum mode with electron beam energies of 10 keV to 20 keV.

## Results and discussion

**Fig. 2:**
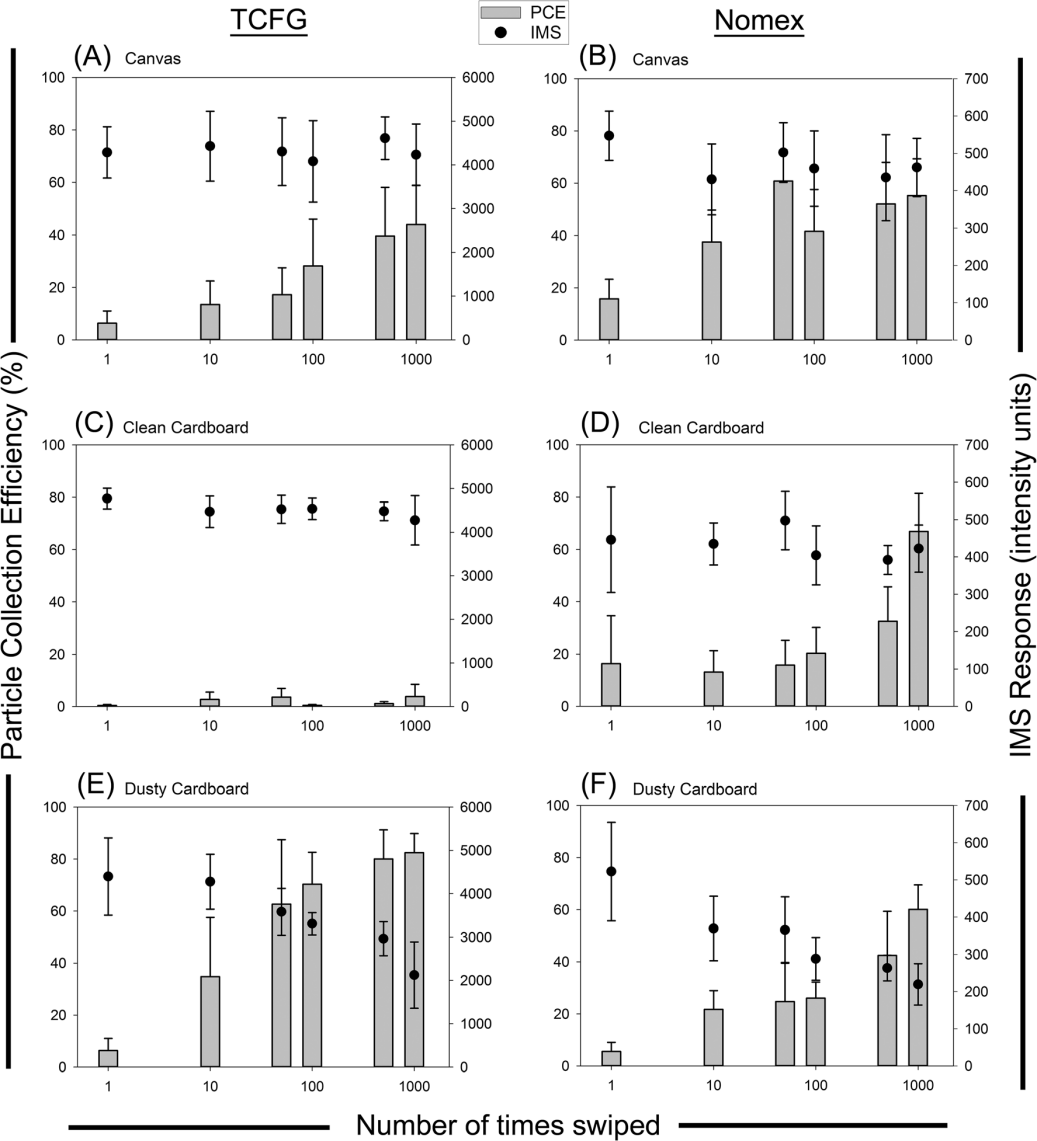
Summary of all PCE and IMS response experiments for reused wipes. The three leftmost plots show results of TCFG swiped on the three different surfaces, while the three rightmost plots represent Nomex. Within each plot, the x-axis is the number of times the wipe was swiped in logarithmic form, the left vertical axis is the particle collection efficiency (PCE), and the right vertical axis is the IMS response in intensity units. *Error bars* are the standard deviation of 10 measurements (one measurement per wipe) for each reuse number

A summary of results for particle collection efficiency and IMS response as a function of the number of times wipes were reused are presented in Fig. 2. Data are segregated into individual plots that display both IMS response and PCE for a single wipe and substrate combination. Within each plot, the x-axis is the wipe reuse number in logarithmic format, the left axis is the PCE from 0 % to 100 %, and the right axis is the IMS response (as intensity units representing the amount of material detected by the instrument). TCFG and Nomex samples were analyzed on different IMS machines, thus the different magnitudes of the right axes. The three leftmost plots (a, c, and e) represent TCFG samples on the three substrates evaluated, while the right plots (b, d, and f) show results from Nomex wipe material. For all plots, bars represent particle collection efficiency (%) and dots represent the IMS response (i.u.), and error bars are 1 standard deviation.

### Repetitive swiping on canvas

The PCE for TCFG wipes repeatedly swiped on the canvas surface (Fig. 2a) increased significantly at a 95 % confidence interval (t-test resulting in a *P* value of 0.0104) as they were reused. Fresh wipes had a PCE of nominally 6 % while wipes that were swiped across a surface 1000 times had a 45 % PCE, an increase by a factor of more than seven. The cause of this dramatic increase is not completely understood, but we speculate that there may be some degree of removal of the Teflon coating on the fiberglass substructure as the wipe is continuously swiped along the surface. Teflon is well known for its non-stick and hydrophobic properties, so removal of this material may result in an increase in the wipes ability to collect material from a surface. It is also possible that that electrostatic charging may play a role in these results, however this was not specifically investigated here. The IMS response remains almost constant as a function of use number, as determined by single factor analysis of variance evaluation for each grouped pair (*F* always less than *F*_*crit*_). Results reveal no clear relationship between number of times wipes were reused and the instrument response for an equal mass of RDX explosive.

**Fig. 3:**
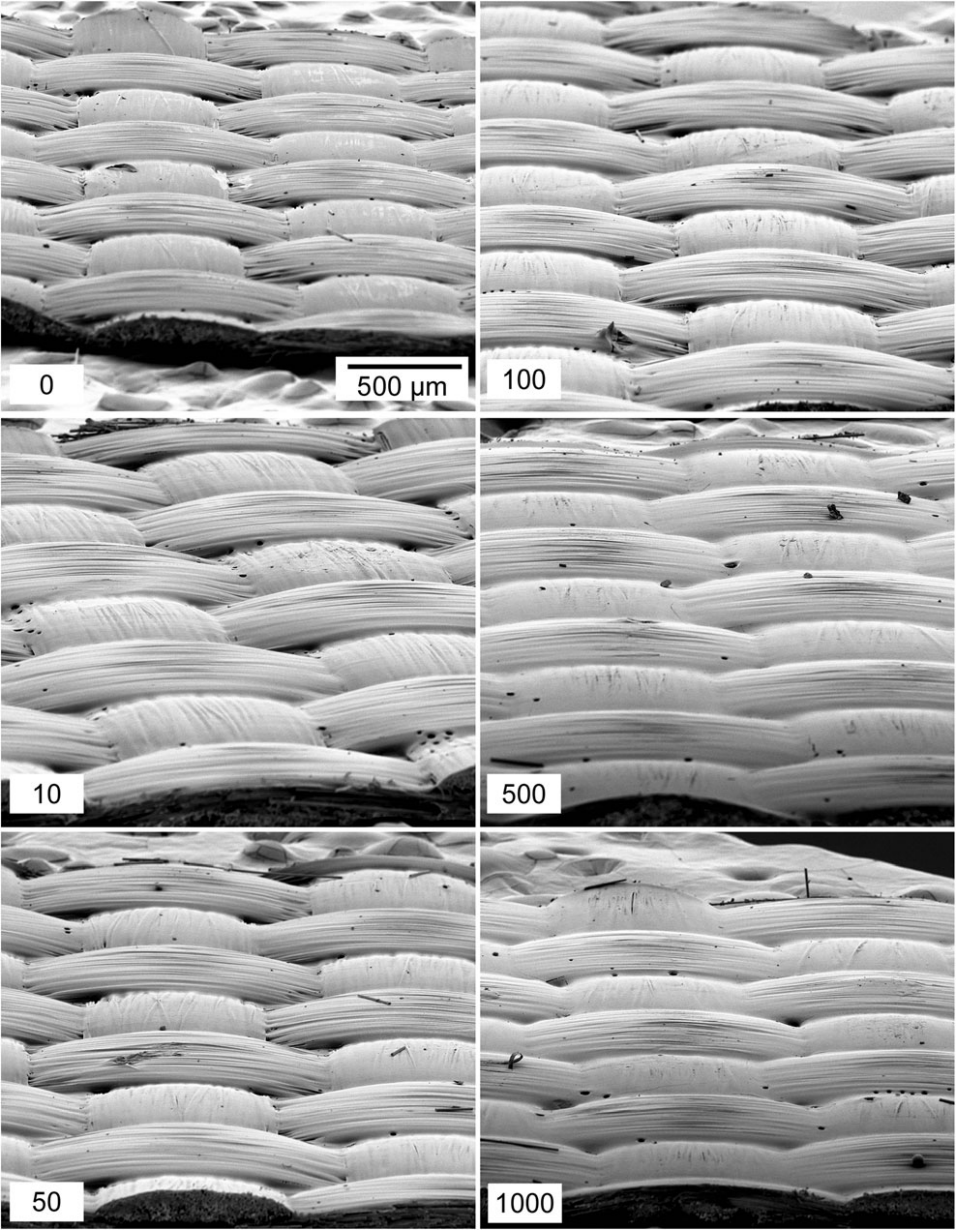
SEM images of TCFG wipes reused on a canvas surface. The bottom left number of each image indicates the number of times the wipe has been reused. The scale bar is 500 μm and each micrograph has nominally the same magnification

A collage of SEM images of TCFG from each reuse number from the canvas surface are shown in Fig. 3. Micrographs of each sample were collected at a high tilt (45°) microscope stage orientation. These images fail to demonstrate a potential removal of the Teflon coating. There was no clear degradation to the bulk surface texture as these wipes are used. Wipes that were used 1000 times appear almost identical to brand new wipes, a surprising result considering the striking improvement in particle collection efficiency with use. Future studies will include looking at these surfaces using other techniques such as atomic force microscopy (AFM) to see if there are changes to the surface on the nano-scale level, which cannot be seen with the SEM used in our study.

Results for Nomex repeatedly swiped on a canvas surface is shown in Fig. 2b. Similar to the results of TCFG repeatedly swiped on canvas, Nomex PCE increased significantly with reuse compared to wipes that were new (t-test resulting in a *P* value of 0.0007, 95 % confidence interval). It appears that the particle collection efficiency reaches a peak once a wipe is used 50 times, and then levels off with increased use. After 50 swipes, the statistical differences with increasing use number are not significant and did not continue to follow a rising trend. The IMS response showed no significant differences for all the wipes regardless of use number as determined with single factor analysis of variance evaluation, however a small suppression in signal was seen in the reused wipes compared to the new wipes.

**Fig. 4:**
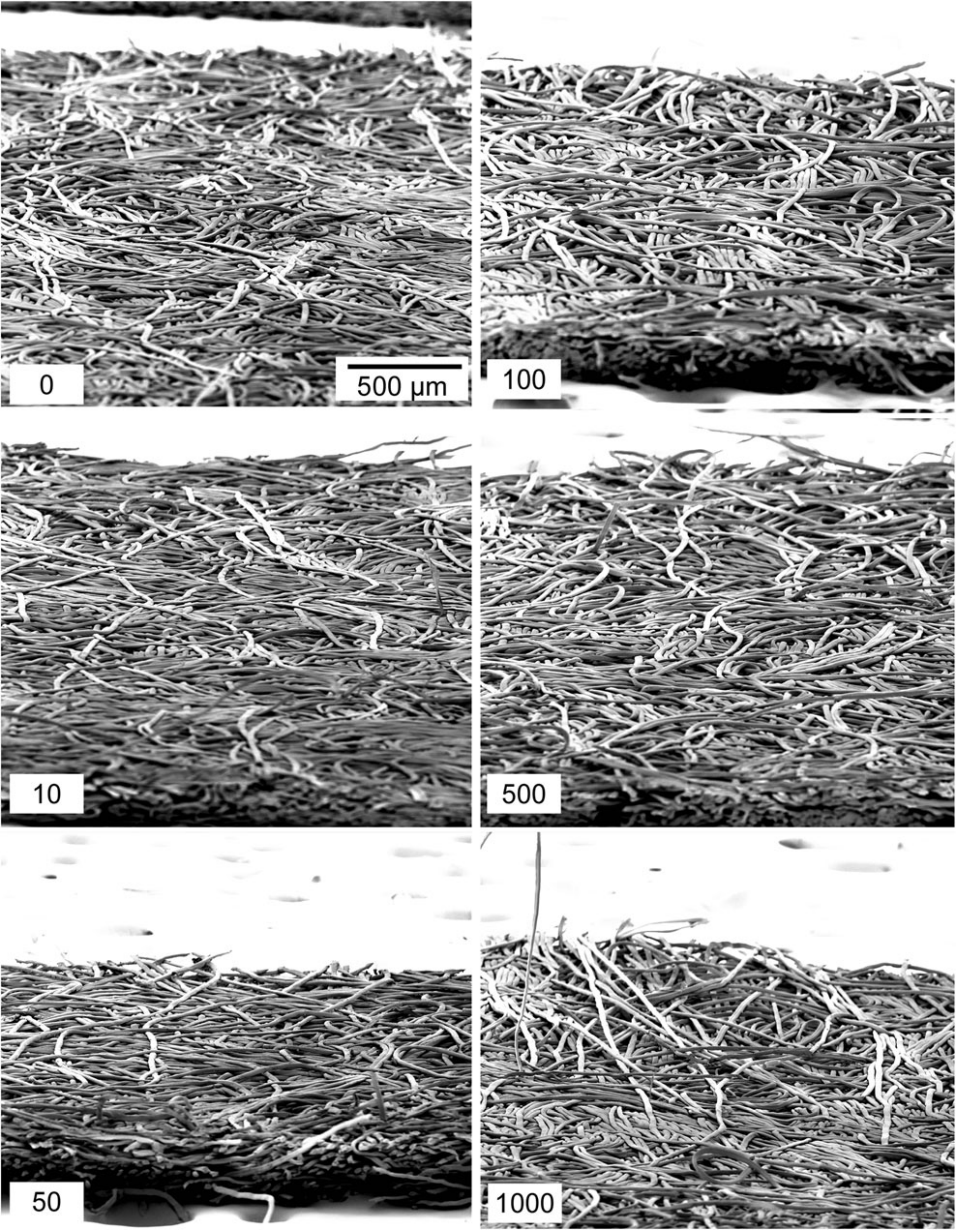
SEM images of Nomex wipes repeatedly swiped on canvas. The bottom left number of each image indicates the number of times the wipe has been used. The scale bar is 500 μm and each micrograph has nominally the same magnification

Figure 4 shows a collage of SEM images for the different number of times Nomex was swiped on the canvas surface. We expected to see indications of fiber surface degradation that would help explain, at least in part, the increase in PCE. Upon close inspection at this magnification there appeared to be no obvious degradation of the weave structure of these materials. This was another surprising result considering the substantial improvement in particle collection efficiency as these wipes were used.

### Repetitive swiping of TCFG wipes on cardboard

**Fig. 5:**
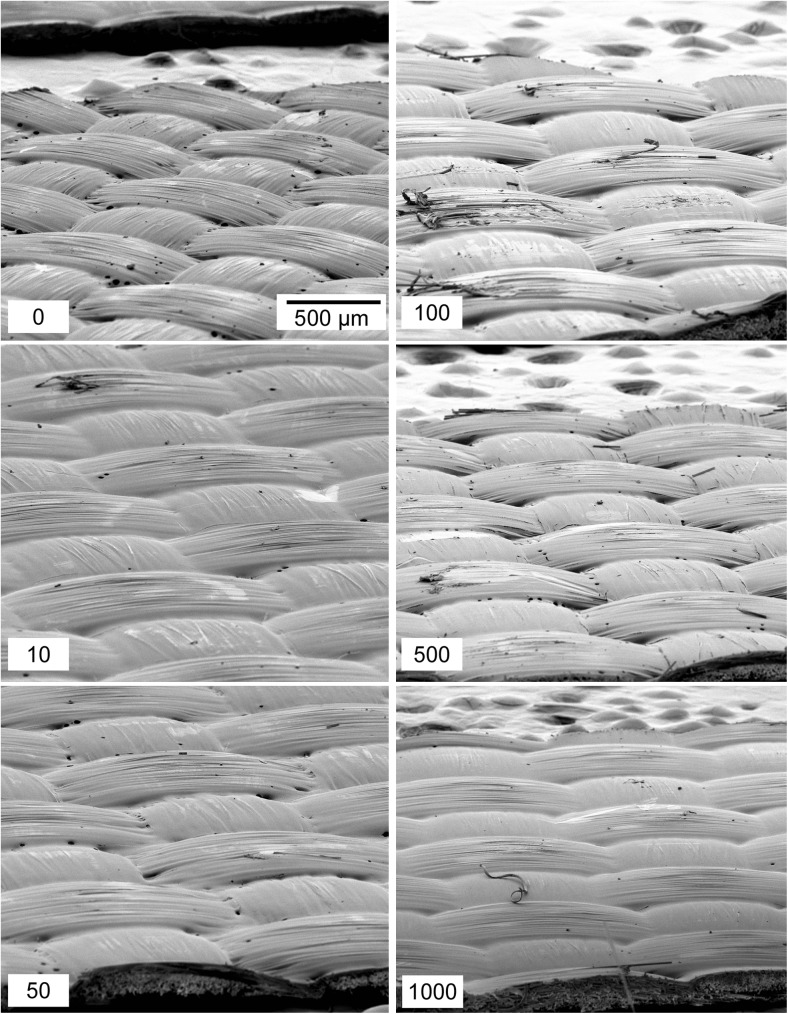
SEM images of TCFG wipes swiped repeatedly on clean cardboard. There are minor abrasions to the surface, but no obvious trend that would suggest surface deterioration with wipe use

The collection efficiencies and IMS results for TCFG wipes swiped on cardboard are shown in Figs. 2c and 2e. For clean cardboard (Fig. 2c), the wipes exhibited insignificant changes in PCE with increasing use number, an expected result considering that these materials demonstrated almost no visible changes in their surface as they were used (see Fig. 5). PCE for these wipes on a clean cardboard surface was quite low, averaging around 2 % collection efficiency. The IMS response of RDX remained almost constant with wipe use number, appearing to have a slight drop in intensity units at 1000 wipes, but this is not a statistically significant difference and is within the deviation of the measurement.

TCFG wipes swiped on dusty cardboard (Fig. 2e) demonstrated a dramatic change with rising wipe use number, with an increase of more than a factor of five between a new wipe and one used ten times. More than a factor of ten increase was shown between a new wipe and one used 1000 times. However this improvement in PCE appeared to come at a cost, as the IMS response for a single mass of RDX was adversely impacted as the use number of the wipe increased. After 100 swipes, the IMS response was reduced 25 % compared to a new wipe. After 1000 swipes, the IMS response was lowered to 50 % of the response of a new wipe. We suspect that this suppression of IMS signal was a result of an increase in unwanted dust being introduced into the IMS chemical detector, possibly interfering with or competing with the chemical ionization process of target analyte molecules within the ionization region of the detector. Mixture compounds and multi-component matrices are known to challenge the selectivity and sensitivity of IMS systems [7, 9, 19], and the dust used in this particular experiment is certainly a multi-component matrix based on the variety of chemical compounds present in the Standard Reference Material. Still, there is a net overall improvement in the efficiency of the wipe since the ability to collect particles exceeds the decrease in IMS response as the use number increases. Although the IMS response was reduced, the instrument was still able to detect relatively low masses of explosive residue.

**Fig. 6:**
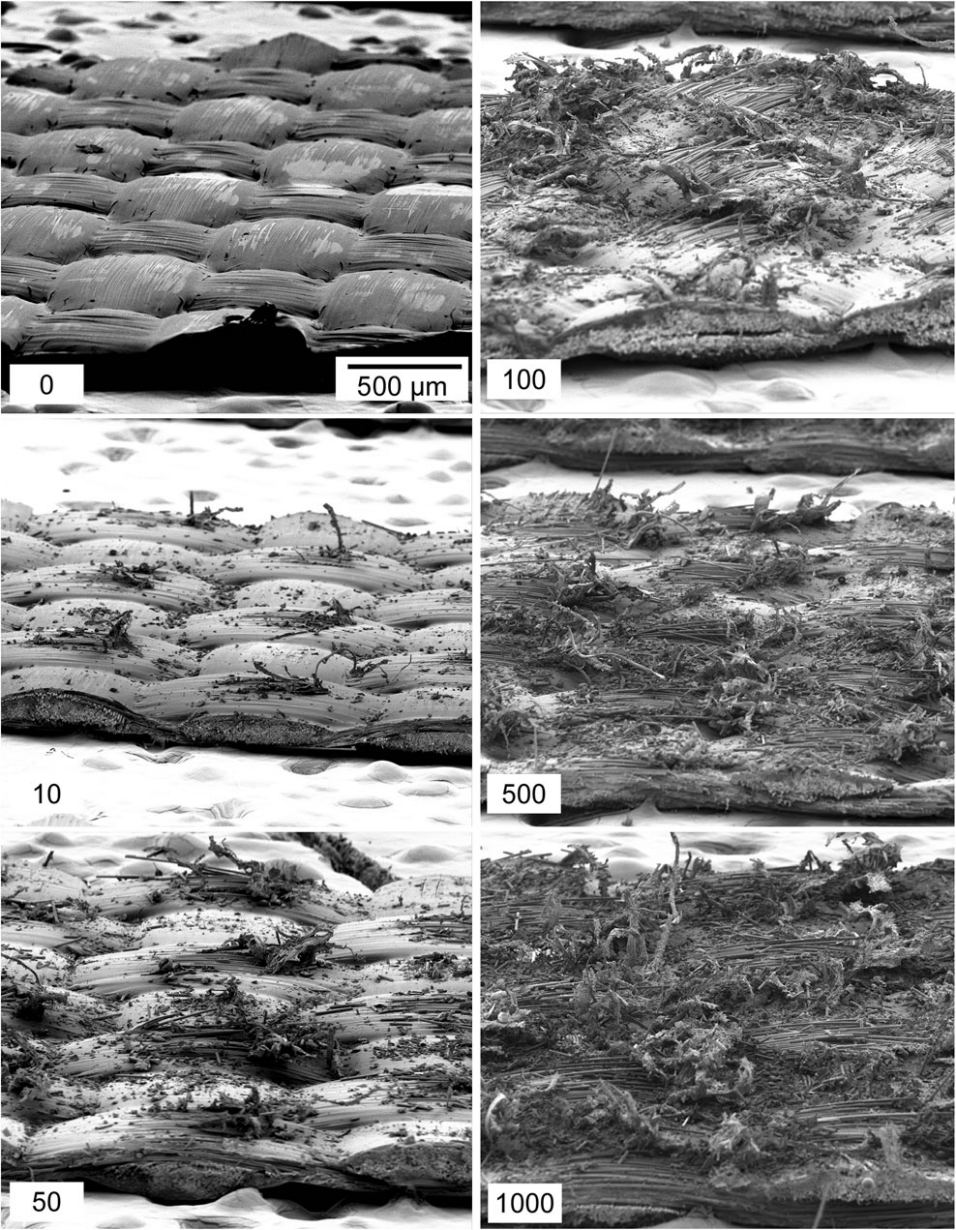
SEM images of TCFG wipes repeatedly swiped on dusty cardboard. The bottom left number on each image indicates the number of times the wipe has been used. Note that even the 10× used you can see the Teflon coating coming off. By 100× used, glass fibers are broken. Both dust particles and the rougher fiberglass surface likely play a role in increasing the PCE of these wipes, however the trade-off must be considered between the enhanced collection ability and reduced chemical response

The suppression of IMS response resulting from the introduction of excess dust is further supported by visualizing the SEM images of TCFG used on dusty cardboard surfaces (Fig. 6). After 50 swipes on the dusty cardboard, the original weave of the TCFG surface began to break apart, exposing and fracturing the fiberglass fibers that create the underlying woven structure. We speculate that the dust acts as an abrasive material that not only roughens the TCFG surface but may also abrade the cardboard substrate, introducing additional particulate matter that interacts during the swiping process. Contrast the images in Fig. 6 with those in Fig. 5, where wipes used on clean cardboard show almost no visible wear.

### Nomex wipes used on cardboard

**Fig. 7:**
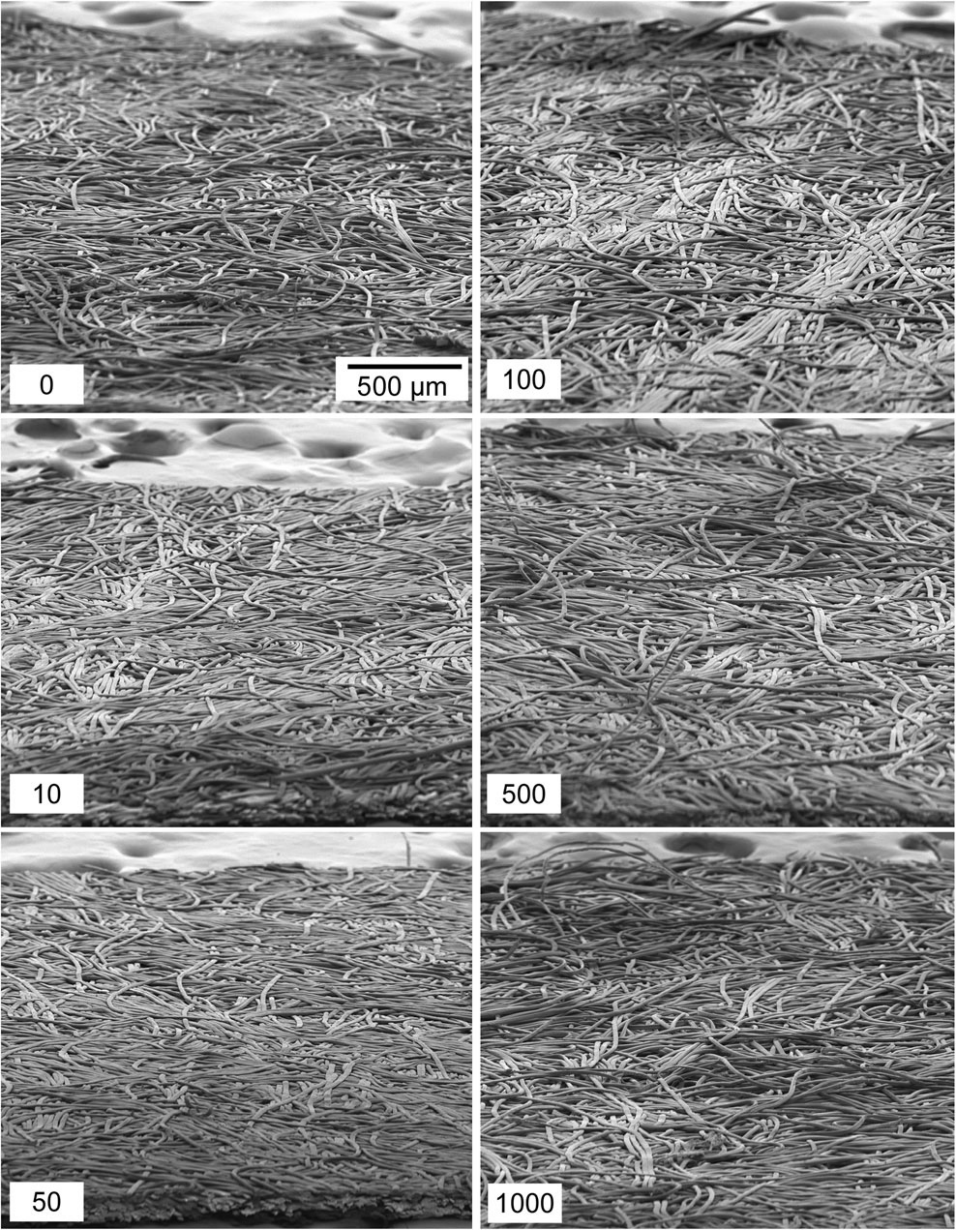
SEM images of Nomex wipes used on clean cardboard. No apparent deterioration of the material is evident as the wipes are used

Results of Nomex used on clean and dusty cardboard surfaces are shown in Figs. 2d and 2f, respectively. The Nomex material exhibited no significant changes in IMS detector response as the number of times of the wipe used on clean cardboard increased (Fig. 2d). However, the particle collection capabilities increased considerably after 500 swipes across the clean cardboard. The PCE at 500 wipes was almost doubled compared to a new wipe, and by 1000 swipes the PCE is nominally four times that of an original wipe. Visualization of the wipes via scanning electron microscopy (Fig. 7) shows that there is little, if any, wear on the surface of these materials, suggesting that the mechanism that is improving the PCE is not necessarily caused by abrasion of the fibrous filaments within the weave of Nomex.

**Fig. 8:**
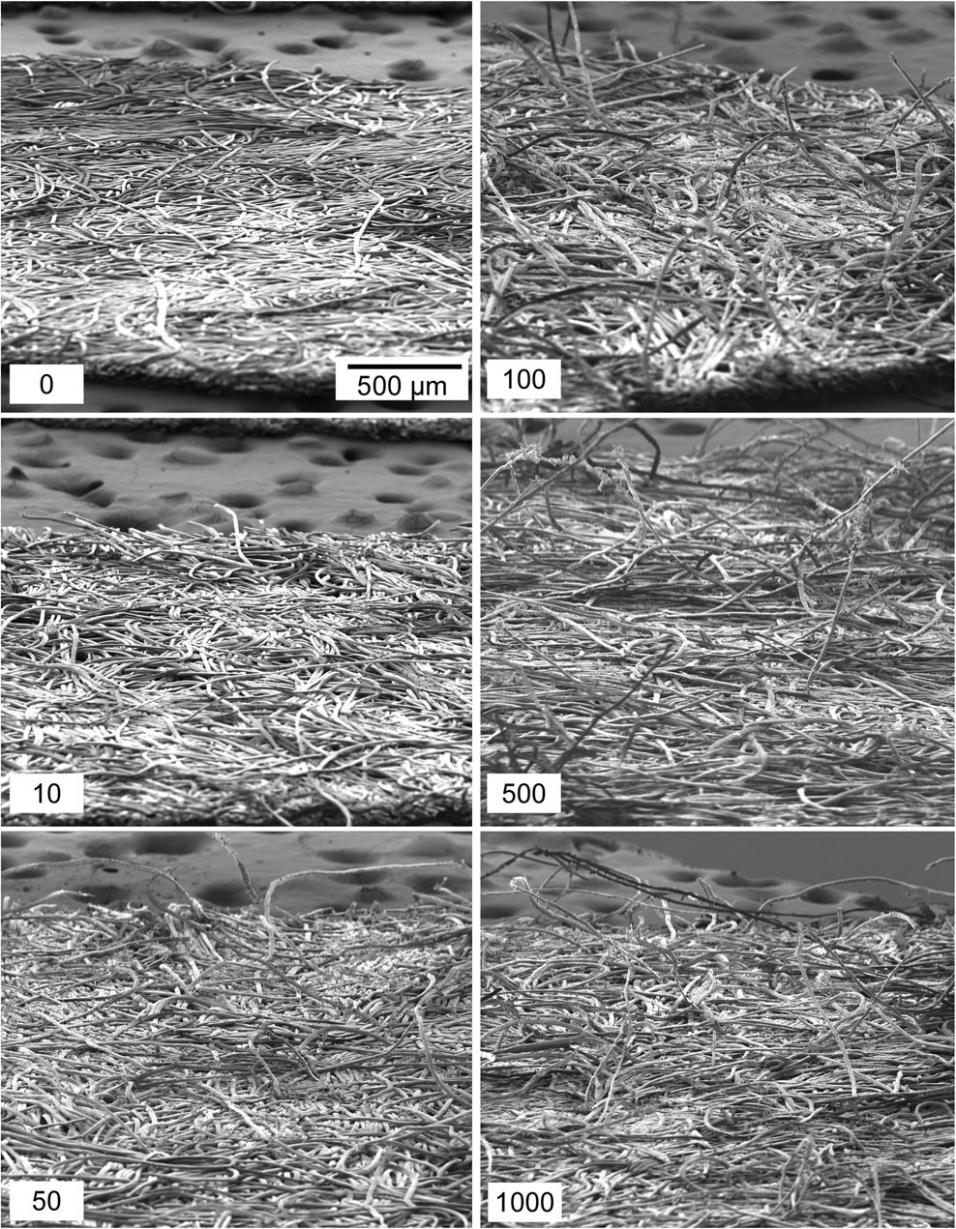
SEM images of Nomex wipes repeatedly swiped on dusty cardboard. The material shows clear deterioration as evidenced by the increasing frequency of stray fibers protruding from the bulk Nomex substrate. Excess visible dirt or dust is not apparent on the surface of the wipes in this example

Nomex swiped on dusty cardboard (Fig. 2f) revealed similar results to those from the TCFG on dusty cardboard, namely an increase in PCE in addition to a suppression of IMS response with use. After just 10 swipes across the dusty cardboard surface, the collection efficiency had increased to nominally 20 %, a four-fold improvement compared to an unused wipe. By 1000 swipes, the Nomex material had a PCE of nearly 60 %. These results are supported by the scanning electron microscopy images found in Fig. 8 that show the progression of Nomex deterioration with increased usage. By 50 swipes, the fibers of the Nomex substrate had begun to lift off of the surface and continued to unravel with additional swipes along the dusty cardboard. Of note for the collage of SEM images in Fig. 8 is the lack of visible dust and debris, contrasted with TCFG on dusty cardboard shown in Fig. 6. Dusty cardboard as the swiping surface with TCFG presented a notable amount of excess dirt on the surface of the wipe as the number of swipes continued to increase. This excess dirt is not present in Fig. 8, implying that surface dust does not interact with Nomex in the same way as TCFG. The dust appeared to enhance the roughening of TCFG to a point where the fibers were completely fragmented, in turn creating additional debris on the bulk wipe surface. For Nomex, the presence of dust only served to loosen the fibers of the wipe and did not act to fragment or damage them. Curiously, the suppression in IMS signal cannot then be solely attributed to the introduction of excess dirt into the IMS detector. The specific cause of the significant reduction in IMS detector response with increased wipe use is unknown and will be the focus of future efforts for this work, which will include using a focused ion beam scanning electron microscope (FIB-SEM) to look at the cross-section of the dusty wipes to see whether dust particles are embedded under the surface.

### Wipe reuse with thermal desorption cycle

Samples analyzed by commercial IMS instruments are typically heated to above 200 °C for seven or more seconds. The wipes cool down fairly quickly after the analysis, and during routine use in the field are often reused as long as there was no alarm (Smith’s Detection; Morpho Detection). The results shown in Fig. 2 do not take into account this thermal cycle of rapid heating and cooling upon chemical analysis. To test the impact of thermal cycling on PCE and IMS response, we repeated some of the experiments for TCFG wipes outlined above but included a heating cycle between each swipe across a surface. A custom robotic arm facilitated transfer of the wipe between the wipe swiping system (see Fig. 1) and the thermal desorber of the Itemiser DX instrument. The robot arm would move the wipe from the surface for swiping to the heated thermal desorber, hold the wipe in the desorber for seven seconds, then remove the wipe from the heater and pause for 10 s to allow the wipe to cool before placing it down on the surface for swiping again. This pattern of swipe – heat – cool was repeated for 10, 50, and 500 times for the TCFG wipes on both canvas and dusty cardboard surfaces.

Results for these additional tests were almost identical to the previous TCFG results, and therefore the data is not shown. For canvas material, the PCE of the Teflon wipes increased while the IMS response stayed steady, similar to what was seen for TCFG and canvas in Fig. 2. Likewise, the TCFG wipes used on dusty cardboard showed an increase in PCE and a decrease in IMS response with increased use. We have concluded that, at least within the experimental parameters used here, thermal cycling of TCFG wipes does not appear to play a significant role in changing IMS response, nor does it accelerate the deterioration of the material.

## Conclusions

This work demonstrates that trace residue collection wipes can be reused multiple times without negatively impacting the overall function of the wipe. Particle collection efficiencies often increased as a function of the number of times the wipe was used. This was especially noticeable with the wipes that collected dust. Examination of the SEM images of the dusty wipes, suggested that the dust helped to abrade the Teflon surface to aid in collecting particles. The Nomex fibers also appeared to unravel. On wipes without dust, the IMS response remained similar as the use of the wipe increased. However we did see a decrease in IMS response as the wipes were swiped on a dusty surface, suggesting that a trade-off between particle collection efficiency and chemical detector response must be considered for trace sampling applications. Table 1 shows an overall summary of the results presented in this work.

**Table 1:** Wipe reuse summary for all experiments

Wipe	Surface	PCE	IMS	SEM
TCFG	Canvas	Increase with use	No significant difference	No visible difference
TCFG	Clean cardboard	No significant difference	No significant difference	No visible difference
TCFG	Dusty cardboard	Significant increase with use	Significant decrease with use	Visibly dirty and abraded
Nomex	Canvas	Variable/increase with use	No significant difference	No visible difference
Nomex	Clean cardboard	Significant increase at high Use	No significant difference	No visible difference
Nomex	Dusty cardboard	Significant increase with use	Significant decrease with use	Visibly dirty

This study does not reflect all aspects of reusing surface sampling wipes, and further work should be done to encompass all aspects of swiping surfaces of interest. Such experiments could include more specific dirt simulants that are common to particular security checkpoint areas, and oily compounds that are often collected during wipe sampling such as fingerprint oils and other sebaceous materials from hands. This work could also be expanded to include collection efficiency measurements using real explosive particles rather than polymer microsphere surrogates.

## Footnotes

1 Certain commercial equipment, instruments, or materials are identified in this document. Such identification does not imply recommendation or endorsement by the National Institute of Standards and Technology, nor does it imply that the products identified are necessarily the best available for the purpose.
